# Investigating the knowledge of and public attitudes towards genetic testing within the Visegrad countries: a cross-sectional study

**DOI:** 10.1186/s12889-020-09473-z

**Published:** 2020-09-10

**Authors:** Klára Bíró, Viktor Dombrádi, Zita Fekete, Gábor Bányai, Klára Boruzs, Attila Nagy, Róza Ádány

**Affiliations:** 1 Department of Health Systems Management and Quality Management for Health Care, Faculty of Public Health, University of Debrecen, Debrecen, Hungary; 2 Department of Behavioural Sciences, Faculty of Medicine, University of Debrecen, Debrecen, Hungary; 3 Faculty of Public Health, University of Debrecen, Debrecen, Hungary; 4 MTA-DE Public Health Research Group, University of Debrecen, Debrecen, Hungary; 5 Department of Public Health and Epidemiology, Faculty of Medicine, University of Debrecen, Debrecen, Hungary

**Keywords:** Genetic testing, Attitudes, Public, Citizens, Visegrad countries

## Abstract

**Background:**

Previous studies have investigated various factors that can determine the attitudes of the citizens considering genetic testing. However, none of them investigated how these attitudes may differ between the Visegrad countries.

**Methods:**

In this cross-sectional study a questionnaire developed by Dutch researchers was translated and used in Hungary, Slovakia, Czechia and Poland. In each country 1000 adult citizens were asked on the topics of personal benefits regarding genetic tests, genetic determinism, and finally, the availability and usage of genetic testing. Multivariate robust regression model was created including several possible influencing factors (such as age, sex, education, marital status, religiousness, and having a genetic test within the nuclear family) to identify the possible differences between the four countries.

**Results:**

The Hungarian citizens had the most positive opinion on the personal benefits of genetic testing followed by the Czech, Slovak and Polish. All differences were significant in this regard. Considering genetic determinism, the Slovak citizens had a significantly firmer belief in this issue compared to the Hungarians. No other significant differences were observed in this domain. On the topic of the availability and use of genetic testing the Hungarian citizens had the most accepting opinion among the four countries, followed by the Czech citizens. In this domain the Polish and Slovak answers did not differ significantly from each other.

**Conclusions:**

Significant differences were observed even when considering various confounding effects. As the underlying reasons for these discrepancies are unknown, future studies should investigate this enigma among the four countries.

## Background

More than 15 years after the completion of Human Genome Project not only health professionals, but also the public have to face the more and more widespread implications of individualised medicine. Rapid development of genome-based diagnostics in the last decades resulted in the growing number of diseases with appropriate genetic testing [1, 2]. Many areas of health services benefit from advantages of precision medicine, and public health is no exception [3–5]. Expanding knowledge on contribution of genetic factors to the development of common chronic diseases (such as cardiovascular, metabolic and malignant diseases) has led to the utilization of information in preventive medicine as preventive genomics [6]. However, no field of preventive medicine can reach its goals without being aware of the factors influencing behaviour of the individuals. One of these factors is attitude. The term attitude refers to a relatively enduring tendency to evaluate an attitude object [7]. Objects of attitudes can be persons, groups, concepts, social policies [8] and – accordingly – medical interventions. The response given on the evaluation can be expressed in affective (emotions and feelings), cognitive (perceptive processes, beliefs) and behavioural (behavioural intentions, behavioural actions) ways [9]. Therefore, a growing body of research is aimed to investigate the beliefs and attitudes of the public towards genetics and genomics in recent years.

These findings are indicating that age, gender and educational level can significantly influence the attitude towards genetic testing. Younger and higher educated women show more accepting attitude [6, 10–12]. These findings suggest that, higher level of education is associated with more knowledge and understanding of genetic testing which can lead to more conforming attitude. According to Rosenstock’s Health Belief Model health-related beliefs and actions partly depend on the knowledge of individuals [13–15] and thus could explain how education level influences attitude. Although, the data of the literature are contradictory. According to some reports more knowledge may result in more scepticism towards genetics [16–18]. Others have not found significant connection between understanding and attitudes [19]. Based on the literature, there is a generally accepting attitude of the pubic towards the genetics used for medical goals [6, 11, 16, 20–22], while a rejective attitude is observed if it is used for producing genetically modified food, cloning animals or studying human embryos [11, 20, 21]. These findings suggest that moral objections may play an important role in formation of attitudes towards genetic testing, where utilizing genetics can be considered as „tampering with nature” [10]. Along with moral issues it is important to mention a further factor that can contribute to the development of attitudes towards genetics, that is genetic determinism. With that term is described the belief that the contribution of genetics to phenotypes is more important than the impact of environmental or social factors [20, 23]. In this regard, according to Parrot et al. religious and spiritual conviction can result in rigid beliefs about genetical determination [24].

As Condit suggests, attitudes towards genetic testing should not be considered as stable or univocal [21]. According to the comparative study between 2002 and 2010 on the Dutch population by Henneman et al. the representation of accepting opinions of the citizens towards the use of genetic achievements in medicine was growing significantly [25]. In addition, according to the results of the Eurobarometer for the year of 2010 52.9% of Europeans showed accepting attitudes towards biotechnology and genetic engineering [26]. However, it is worth mentioning, that the results came from a single question, and did not go into the different aspects of genetic screening.

Therefore, to get up-to-date information and to understand better the citizens’ attitude towards genetic testing, an international survey was conducted in the countries of the Visegrad group, i.e. in Hungary, Slovakia, Czechia and Poland (collectively known as the V4 countries) [26, 27]. These countries cooperate closely for decades and in the past few years, several studies were conducted that compared them to one another [27–30]. Thus, the objective of this study was to investigate the attitudes of the citizens within the Visegrad countries towards genomic testing and to explore the possible differences among them.

## Methods

### Design, setting and respondents

A questionnaire based international cross-sectional study was conducted in Hungary, Slovakia, Czechia and Poland. Private polling companies were contracted to collect data within the four countries, in Hungary the SZLEM Service L. P., while in Slovakia, Czechia and Poland the Český Národní Panel Ltd. In every country, the same study design and instrument were applied. The request was made to the companies to fill out the questionnaire with 1000 citizens per each country between the age of 18 and 65. The data collection was executed on an online market research panel, in which the panel members preliminary gave their consent for the data processor to handle personal data for the purpose of inviting them to market researches. In all countries the panels include citizens of the country from all demographic groups representing the whole population, 17,000–50,000 members [31]. These were the citizens to which the private polling companies could send the questionnaires. The respondents were gradually invited via individual emails, using crosslinked quotas to ensure representative sampling by age (18–65), sex, number of residents living in the settlement, and region. The quotas for the layered samples were monitored by an automatic data collection information technology system ensuring the valid ratio of each subsample. This means, that if – for example – the elderly started to become overrepresented in the sample compared to the overall population, then the system only sent invitations randomly to those being younger. The process of invitation lasted until they have reached 1000 respondents for each country.

Before the questionnaires were sent out ethical approval had been obtained in those countries in which non-intervention studies require one. In each country the online survey was conducted from May to June 2018. Answering every question was made mandatory for the participants.

### Measures

Parts of a questionnaire was used in this study which was developed by Henneman and her colleagues [10]. The original questionnaire asked a wide variety of questions regarding application of genetics and genetic testing in healthcare and several studies were conducted with this instrument [6, 10, 25, 32]. Since this questionnaire was designed to assess general opinion on genetic testing, the questions did not specify what kind of genetic testing, for what purpose, and in what circumstances should be considered. For each of the questions an answer could be given on a 5-point Likert scale in which a 1 meant that the respondent completely disagrees with the statement and a 5 meant complete agreement. Following the method used in a previous Dutch study three domains were created and analysed: belief in personal benefits, belief in genetic determinism, and availability and use of genetic tests [10]. The questions regarding the demographic data, such as marital status or being religious, were entirely adopted from the original Dutch questionnaire as well [10]. Permission was granted by the developer of the questionnaire to translate and use it for research purposes.

Identical practice guide was used for translating and adapting the questionnaire for Hungarian, Slovak, Czech and Polish languages. First, two independent translators translated the English questionnaire into the target language. Then, based on the quality of the translations, the two were merged into a single one [33]. In order to test how understandable and grammatically correct is the translation ten citizens were asked to evaluate the questionnaire. Based on their comments the questionnaire was modified. The final version of the translation was translated back into English with a third independent translator.

### Data analyses

While conducting the descriptive data analyses for the characteristics of the respondents and for the given answers to each question the answers of the four countries were not aggregated and were treated independently from one another. The internal reliability of each domain was tested with Cronbach’s alpha. The alpha value was deemed acceptable if it was equal or above 0.60 [34]. The value of the domain was calculated by adding up the values linked to responses on the Likert scale. For the personal benefits, and for the availability and use of genetic tests domains, higher values reflected a more accepting attitude towards genetic testing. As the questions within the genetic determinism domain focused on the negative aspects of genetic testing, getting higher scores indicated a more rejecting attitude.

The validity of the four translated questionnaires were assessed with confirmatory factor analysis. For this aim Comparative Fit Index (CFI), Tucker-Lewis Index (TLI), Standardized Root Mean Square Residual (SRMR), and Root Mean Square Error of Approximation (RMSEA) were used and the thresholds were set based on the literature [35–37].

The originally continuous data of age was dichotomized in order to be used in the same manner as the other demographic data. In accordance with the aforementioned Dutch studies the threshold for the dichotomizing process was set at 39 [6, 10, 25, 32]. For the comparative analyses robust regression was applied. Besides comparing each country to another we also investigated how the various demographic data (such as sex, age, education level, marital status, being religious), and having a genetic test among the family in the past (self, partner or child) influence the citizens opinion regarding the belief in personal benefits of genetic tests, the belief in genetic determinism, and the availability and usage of genetic tests. During the multivariate analyses the variables were selected for inclusion when they are with biological or plausible rationale, or when the univariate analysis results in a *P*-value of ≤0.20 [38]. A *P*-value less than 0.05 was considered significant. For all the statistical analyses Intercooled Stata 10.0 was used.

## Results

### Sample characteristics

The response rate of the panels varied from 45 to 60%. This means that the companies had to send around 2000 emails per country to get the 1000 response at the end of the survey. Once the survey was completed, overall, 4000 responses (1000 from each participating country) were analysed. The characteristics of these participants are shown in Table 1. In each country female respondents were in slight majority (50.5–52.1%). Most of the participants were 40–65 years old (53.3–71.7%). Also, the majority of them were married (53.5–65.0%). Only 38.6% of Czechs and 61.9% of Hungarians were religious in some extent (not stating being non-religious) compared to 82.7% in Slovakia and 91.4% in Poland. Furthermore, while only 7.4% of Poles stated that someone within the nuclear family (self, partner or children) had a genetic test before, 11.2% of Hungarians, 15.0% of Slovaks and a noteworthy 21.1% of Czechs made the same statement. Regarding education, while around half of the Czech, Polish and Slovak citizens only had a primary or secondary vocational education (44.0–55.0%), this ratio was 34.6% among the Hungarians.

**Table 1:** Characteristics of the respondents involved in the survey

	Hungary		Slovakia		Czechia		Poland	
	n	%	n	%	n	%	n	%
**Sex**								
Male	495	49.5%	492	49.2%	490	49.0%	479	47.9%
Female	505	50.5%	508	50.8%	510	51.0%	521	52.1%
**Age**								
18–24	109	10.9%	131	13.1%	120	12.0%	130	13.0%
25–39	286	28.6%	352	35.2%	336	33.6%	337	33.7%
40–65	605	60.5%	517	51.7%	544	54.4%	533	53.3%
**Education**								
Primary or secondary vocational school	346	34.6%	550	55.0%	491	49.1%	440	44.0%
High school	421	42.1%	341	34.1%	342	34.2%	360	36.0%
College or university	233	23.3%	109	10.9%	167	16.7%	200	20.0%
**Marital status**								
Married	607	60.7%	560	56.1%	535	53.5%	650	65.0%
Not married, divorced	361	36.1%	414	41.4%	451	45.1%	309	30.9%
Widow	32	3.2%	25	2.5%	14	1.4%	41	4.1%
**Religious**								
Very actively religious	103	10.3%	188	18.8%	46	4.6%	294	29.4%
Actively religious	232	23.2%	454	45.4%	160	16.0%	298	29.8%
Not actively	284	28.4%	185	18.5%	180	18.0%	322	32.2%
Not religious	381	38.1%	173	17.3%	614	61.4%	86	8.6%
**Previous genetic test** **(self, partner, children)**								
Yes	112	11.2%	150	15.0%	211	21.1%	74	7.4%
No	878	87.8%	850	85.0%	789	78.9%	926	92.6%

### Internal reliability and validity

The responses for the individual questions are presented in Table 2, while the aggregated scores for the domains are shown in Table 3. The internal reliability was above 0.70 in all domains and for all countries, with the exception of the availability and use of genetic tests domain for Slovakia (alpha = 0.68). Regarding the confirmatory factor analyses the CFI value was 0.878 in Hungary, 0.894 in Slovakia, 0.897 in Czechia, and 0.928 in Poland. The TLI values were somewhat similar, with 0.851 in Hungary, 0.871 in Slovakia, 0.875 in Czechia, and 0.912 in Poland. The SRMR value of the Hungarian translation was 0.156, 0.118 for the Slovak, 0.159 for the Czech, and 0.170 for the Polish. Finally, the RMSEA was 0.092 in Hungary, 0.082 in Slovakia, 0.094 in Czechia, and 0.087 in Poland.

**Table 2:** Means, standard deviations, medians and interquartile ranges of the responses regarding the attitudes towards genetic testing

Items	Hungary				Slovakia				Czechia				Poland			
	Mean	SD	Q2	IQR	Mean	SD	Q2	IQR	Mean	SD	Q2	IQR	Mean	SD	Q2	IQR
**Personal benefits**																
To prevent disease I would want to know my risk of getting certain diseases	3.81	1.28	4	2	3.54	1.28	4	2	3.84	1.25	4	2	3.38	1.37	4	3
I am curious about my genetic make-up	3.66	1.36	4	2	3.34	1.22	3	1	3.46	1.2	4	1	3.36	1.37	3	3
I do not want to know what kind of diseases I could get in the future^a^	3.44	1.41	4	3	3.34	1.24	3	1	3.51	1.24	4	2	3.39	1.37	3	3
Knowing my risk of getting a serious disease, I would be able to control my life more	3.66	1.26	4	2	3.38	1.1	3	1	3.61	1.16	4	2	3.31	1.28	3	2
**Genetic determinism**																
When people know their genetic make-up they will not be able to lead their own lives	2.78	1.26	3	2	2.86	1.11	3	2	2.66	1.13	3	1	2.75	1.23	3	2
When people know their genetic make-up they will take less responsibilities	2.47	1.21	2	2	2.55	1.05	3	1	2.67	1.06	3	1	2.71	1.18	3	1
People’s knowledge of their genetic make-up will decrease their self-confidence	2.88	1.23	3	2	2.85	1.05	3	1	2.75	1.07	3	1	2.76	1.21	3	2
Genetic tests deprive people’s freedom to live as they want	2.59	1.3	3	3	2.87	1.13	3	1	2.72	1.15	3	1	2.85	1.28	3	2
**Availability and use of genetic tests**																
The use of genetic tests among people should be stimulated	3.26	1.24	3	1	2.83	0.98	3	1	3.49	1.09	3	1	3.22	1.18	3	1
Genetic tests should be available for those who want to use them	4.07	1.22	5	2	3.62	1.19	4	2	3.97	1.19	4	2	3.59	1.31	4	2
More money should be available for the development of genetic tests	3.98	1.16	4	2	3.36	1.1	3	1	3.57	1.05	4	1	3.34	1.25	3	1
Genetic tests should be offered to all pregnant women	4.15	1.14	5	2	3.64	1.17	4	2	3.71	1.16	4	2	3.41	1.29	3	2

^a^In the scale analyses the answers were recoded (1 = 5; 2 = 4; 4 = 2; 5 = 1). SD: Standard deviation; Q2: Median; IQR: Interquartile range

**Table 3:** Internal reliability and descriptive characteristics of the main domains regarding the attitudes towards genetic testing

	Cronbach’s alfa	Mean	SD	Q1	Median	Q3	IQR
**Personal benefits**							
Hungary	0.815	3.64	1.07	3.00	3.75	4.50	1.50
Slovakia	0.817	3.40	0.97	2.75	3.50	4.00	1.25
Czechia	0.830	3.60	0.99	3.00	3.75	4.25	1.25
Poland	0.868	3.36	1.14	2.75	3.50	4.25	1.50
**Genetic determinism**^**a**^							
Hungary	0.813	2.68	1.00	2.00	2.75	3.25	1.25
Slovakia	0.774	2.78	0.84	2.25	3.00	3.25	1.00
Czechia	0.827	2.70	0.90	2.00	2.75	3.25	1.25
Poland	0.840	2.77	1.01	2.00	3.00	3.25	1.25
**Availability and use of genetic tests**							
Hungary	0.785	3.87	0.93	3.25	4.00	4.50	1.25
Slovakia	0.682	3.36	0.79	3.00	3.25	4.00	1.00
Czechia	0.818	3.68	0.90	3.00	3.75	4.50	1.50
Poland	0.862	3.39	1.06	2.75	3.50	4.25	1.50

^a^The questions of genetic determinism are focused on the negative aspects of genetic testing

*SD* Standard deviation, *Q1* Lower quartile, *Q3* Upper quartile, *IQR* Interquartile range

### Attitudes towards genetic testing

As seen in Table 3 the highest mean for personal benefits was for Hungary with 3.64 (SD = 1.07), followed by Czechia (mean = 3.60; SD = 0.99), Slovakia (mean = 3.40; SD = 0.97) and Poland (mean = 3.36; SD = 1.14). Also, Hungarians had the lowest scores for genetic determinism (mean = 2.68; SD = 1.00), while Czech (mean = 2.70; SD = 0.90), Polish (mean = 2.77; SD = 1.01) and Slovak (mean = 2.78; SD = 0.84) respondents had higher scores. Similarly, Hungarian citizens support the availability and usages of genetic testing the most (mean = 3.87; SD = 0.93), than Czech (mean = 3.68; SD = 0.90) and Polish citizens (mean = 3.39; SD = 1.06), while Slovak participants were the least supportive (mean = 3.36; SD = 0.79).

### Investigating the demographic factors

The results of the robust regression analyses as univariate model and as multivariate model for the personal benefits domain is shown in Table 4, for the genetic determinism domain in Table 5, and considering the domain of the availability and usage of genetic tests in Table 6.

**Table 4:** Multivariate analysis of personal benefits within the Visegrad countries

Factors		Univariate analysis				Multivariate analysis			
		β	P-value	95% CI		β	P-value	95% CI	
Sex	Female/**Male**	0.29	< 0.001*	0.14	0.44	0.27	< 0.001*	0.12	0.42
Age	40–65/**18–39**	0.01	0.035*	0.001	0.01	0.01	0.006*	0.003	0.02
Education	High school/ **Primary or vocational secondary**	0.49	< 0.001*	0.32	0.66	0.39	< 0.001*	0.23	0.56
	Higher/ **Primary or vocational secondary**	0.63	< 0.001*	0.43	0.83	0.49	< 0.001*	0.28	0.70
Marital status	Married/**Other**	−0.07	0.364	−0.22	0.08	−0.14	0.067	−0.30	0.01
Religious	Yes/**No**	− 0.49	< 0.001*	− 0.65	− 0.33	0.09	0.004*	0.03	0.15
Genetic testing (self, partner, children)	Yes/**No**	−0.34	0.002*	−0.56	− 0.12	− 0.28	0.013*	− 0.49	− 0.06
Hungary(HUN)	SK/**HUN**	− 0.76	< 0.001*	− 0.97	− 0.55	− 0.57	< 0.001*	− 0.78	−0.35
	CZ/**HUN**	−0.28	0.010*	−0.48	− 0.07	− 0.24	0.028*	− 0.46	− 0.03
	PL/**HUN**	−1.02	< 0.001*	−1.23	− 0.81	− 0.83	< 0.001*	−1.04	− 0.61
Slovakia(SK)	HUN/**SK**	0.76	< 0.001*	0.55	0.97	0.57	< 0.001*	0.35	0.78
	CZ/**SK**	0.49	< 0.001*	0.28	0.70	0.32	0.005*	0.10	0.54
	PL/**SK**	−0.26	0.016*	−0.47	− 0.05	− 0.26	0.016*	− 0.47	− 0.05
Czechia(CZ)	HUN/**CZ**	0.28	0.010*	0.07	0.48	0.24	0.028*	0.03	0.46
	SK/**CZ**	−0.49	< 0.001*	−0.70	− 0.28	−0.32	0.005*	−0.54	− 0.10
	PL/**CZ**	−0.74	< 0.001*	−0.95	− 0.53	− 0.58	< 0.001*	− 0.81	−0.35
Poland(PL)	HUN/**PL**	1.02	< 0.001*	0.81	1.23	0.83	< 0.001*	0.61	1.04
	SK/**PL**	0.26	0.016*	0.05	0.47	0.26	0.016*	0.05	0.47
	CZ/**PL**	0.74	< 0.001*	0.53	0.95	0.58	< 0.001*	0.35	0.81

*β* Beta coefficient, *CI* Confidence interval, *P* Significance of statistical test; Reference groups are in bold; *Significant findings (*P* < 0.05)

**Table 5:** Multivariate analysis of genetic determinism within the Visegrad countries

Factors		Univariate analysis				Multivariate analysis			
		β	P-value	95% CI		β	P-value	95% CI	
Sex	Female/**Male**	−0.02	0.879	−0.25	0.21	0.02	0.859	−0.21	0.26
Age	40–65/**18–39**	0.01	0.012*	0.003	0.02	0.01	0.062	−0.001	0.02
Education	High school/ **Primary or vocational secondary**	−0.17	0.204	−0.43	− 0.09	− 0.11	0.400	− 0.38	0.15
	Higher/ **Primary or vocational secondary**	−0.26	0.106	−0.58	− 0.55	− 0.16	0.333	− 0.49	0.17
Marital status	Married/**Other**	0.27	0.025*	0.03	0.51	0.29	0.021*	0.04	0.53
Religious	Yes/**No**	0.32	0.013*	0.07	0.57	−0.08	0.104	−0.17	0.02
Genetic testing (self, partner, children)	Yes/**No**	0.85	< 0.001*	0.51	1.18	0.89	< 0.001*	0.54	1.23
Hungary(HUN)	SK/**HUN**	0.41	0.014*	0.09	0.74	0.39	0.025*	0.05	0.74
	CZ/**HUN**	0.08	0.617	−0.24	0.41	0.23	0.196	−0.12	0.57
	PL/**HUN**	0.35	0.035*	0.02	0.68	0.23	0.186	−0.11	0.57
Slovakia(SK)	HUN/**SK**	−0.41	0.014*	−0.74	−0.09	− 0.39	0.025*	− 0.74	−0.05
	CZ/**SK**	−0.33	0.049*	−0.66	− 0.001	− 0.17	0.355	− 0.52	0.19
	PL/**SK**	−0.06	0.716	−0.39	−0.27	− 0.16	0.341	− 0.49	0.17
Czechia(CZ)	HUN/**CZ**	−0.08	0.617	− 0.41	0.24	−0.23	0.196	−0.57	0.12
	SK/**CZ**	0.33	0.049*	0.001	0.66	0.17	0.355	−0.19	0.52
	PL/**CZ**	0.27	0.109	−0.06	0.60	0.005	0.980	−0.36	0.37
Poland(PL)	HUN/**PL**	−0.35	0.035*	−0.68	−0.02	− 0.23	0.186	− 0.57	0.11
	SK/**PL**	0.06	0.716	−0.27	0.39	0.16	0.341	−0.17	0.49
	CZ/**PL**	−0.27	0.109	−0.6	0.06	−0.005	0.980	−0.37	0.36

*β* Beta coefficient, *CI* Confidence interval, *P* Significance of statistical test; Reference groups are in bold; *Significant findings (*P* < 0.05)

**Table 6:** Multivariate analysis of use of genetic tests within the Visegrad countries

Factors		Univariate analysis				Multivariate analysis			
		β	P-value	95% CI		β	P-value	95% CI	
Sex	Female/**Male**	0.78	< 0.001*	0.54	1.01	0.77	< 0.001*	0.54	1.00
Age	40–65/**18–39**	0.02	< 0.001*	0.01	0.03	0.02	< 0.001*	0.01	0.03
Education	High school/ **Primary or vocational secondary**	0.81	< 0.001*	0.55	1.07	0.54	< 0.001*	0.28	0.80
	Higher/ **Primary or vocational secondary**	1.17	< 0.001*	0.85	1.49	0.73	< 0.001*	0.41	1.05
Marital status	Married/**Other**	0.13	0.299	−0.11	0.36	−0.04	0.733	−0.28	0.20
Religious	Yes/**No**	−1.00	< 0.001*	−1.25	−0.74	0.20	< 0.001*	0.11	0.29
Genetic testing (self, partner, children)	Yes/**No**	−0.45	0.010*	−0.79	− 0.11	−0.34	0.046*	−0.68	− 0.01
Hungary(HUN)	SK/**HUN**	−2.01	< 0.001*	−2.34	− 1.69	− 1.66	< 0.001*	− 1.99	− 1.32
	CZ/**HUN**	−0.73	< 0.001*	− 1.05	− 0.40	−0.70	< 0.001*	− 1.04	−0.37
	PL/**HUN**	− 1.9	< 0.001*	− 2.22	− 1.58	−1.56	< 0.001*	− 1.89	− 1.22
Slovakia(SK)	HUN/**SK**	2.01	< 0.001*	1.69	2.34	1.66	< 0.001*	1.32	1.99
	CZ/**SK**	1.28	< 0.001*	0.96	1.61	0.95	< 0.001*	0.61	1.30
	PL/**SK**	0.11	0.499	−0.21	0.44	0.10	0.543	−0.22	0.43
Czechia(CZ)	HUN/**CZ**	0.73	< 0.001*	0.40	1.05	0.70	< 0.001*	0.37	1.04
	SK/**CZ**	−1.28	< 0.001*	−1.61	−0.96	−0.95	< 0.001*	− 1.30	− 0.61
	PL/**CZ**	−1.17	< 0.001*	−1.50	−0.85	−0.85	< 0.001*	− 1.21	−0.49
Poland(PL)	HUN/**PL**	1.90	< 0.001*	1.58	2.22	1.56	< 0.001*	1.22	1.89
	SK/**PL**	−0.11	0.499	−0.44	0.21	−0.10	0.543	−0.43	0.22
	CZ/**PL**	1.17	< 0.001*	0.85	1.50	0.85	< 0.001*	0.49	1.21

*β* Beta coefficient, *CI* Confidence interval, *P* Significance of statistical test; Reference groups are in bold; *Significant findings (*P* < 0.05)

Regarding personal benefits (Table 4), while considering confounding effects females had a more accepting opinion than males (*P* < 0.001). Also, the participants between the age of 40–65 had a more favourable view than those between the age of 18–39 (*P* = 0.006). Those having a higher or a high school education had also a more accepting view than those with a primary or a secondary vocational education (*P* < 0.001 in both cases). Religious participants (*P* = 0.004) or those who had genetic test within the nuclear family (self, partner or children; *P* = 0.013) had a more rejecting view on this issue. Those being married (*P* = 0.021) and those who had genetic testing within the family (self, partner or children; *P* < 0.001) believed more in genetic determinism (Table 5). On the topic of using genetic tests (Table 6) female participants, as well as the ones between age of 40–65 had more accepting view (*P* < 0.001). Participants with higher or high school had a more favourable view on the usage of genetic tests than those with primary or secondary vocational education (*P* < 0.001 in both cases). Those who claimed to be somewhat religious (*P* < 0.001) and those who had genetic testing within the family (self, partner or children; *P* = 0.046) had a more negative view on this topic.

### Comparing the Visegrad countries to one another

While considering confounding effects, regarding the domain of belief in personal benefits (Table 4) both Hungarian and Czech respondents had a significantly more accepting attitude than Slovak and Polish citizens (*P* < 0.001 in both cases). When comparing the Hungarian and Czech responses, the Hungarians had a significantly more positive opinion (*P* = 0.028; β = 0.24). Between the Slovak and Polish participants significant difference was also identified (*P* = 0.016) in which the Slovaks had a more positive opinion compared to the Poles (β = 0.26). Regarding the citizens belief in genetic determinism (Table 5), the Slovak respondents had a significantly firmer belief in this compared to the Hungarians (*P* = 0.025; β = 0.39). No other significant difference was observed in this domain. Finally, considering the attitude on the availability and use of genetic tests (Table 6) Hungarian citizens were the most inclusive compared to Czech, Polish and Slovak respondents (*P* < 0.001 in all cases). Czech participants were significantly more conforming compared to both the Polish and Slovak respondents (*P* < 0.001 in both cases). However, no significant difference was found between the Slovak and Polish opinions (*P* = 0.543).

## Discussion

This study was conducted in order to explore the citizen’s attitudes towards genetic testing within the Visegrad countries and to investigate the differences between these countries. When assessing the overall opinions, the results obtained in the Dutch survey conducted in 2010 can be used as reference since the questions were identical [25]. However, it is worth emphasising that the methodology of the survey was somewhat different and the eight-year difference in timing should also be considered.

While 10% of Dutch citizens reported having a genetic test for themselves, their partner or their child(ren) in 2010 [25], only 7.4% of Polish respondents replied the same answer in our survey. Because more than 10% of the citizens of the other three countries replied the same, it can be assumed that Poland lags behind in the usage of genetic testing, especially compared to the 21.1% Czech response.

Generally, the mean scores were more favourable for the Visegrad countries in 2018 than that of the Dutch respondents in 2010. While the mean score of answers to the ‘I am curious about my genetic testing’ question for the Dutch was 3.17 (SD = 1.25) [25], all the Visegrad countries had a mean above 3.34. Furthermore, the Dutch mean regarding the question ‘Genetic tests deprive people’s freedom to live as they want’ was 3.02 (SD = 1.20) [25], while the highest, and thus the worst mean was only 2.87 for the Slovak citizens. However, there were a few questions in which the 2010 Dutch response was more accepting than of the citizens of some of the Visegrad countries: regarding the ‘To prevent diseases I would like to know my risk of getting certain disease’ statement the mean score for the Polish was lower than that of the Dutch (mean = 3.46; SD =1.25); for the ‘Genetic tests should be available for those who want to use them’ question both Slovak and Polish means were lower (Dutch mean = 3.66; SD = 1.27), and for the ‘More money should be available for the development of genetic tests’ statement only the Hungarian mean score was higher than the Dutch (mean = 3.61; SD =1.07). Overall, it can be stated that the citizens of the Visegrad countries have a relatively more accepting view on genetic testing; however, there are some specific issues where it is more rejective compared to the 2010 Dutch survey. The possibility that these differences became even higher in 2018 cannot be excluded considering the eight-year gap.

In accordance with the literature, sex, age and education level significantly impacted the opinion on both the personal benefits and the usage of genetic testing [16–18]. However, while previous studies have shown that younger people have a more positive opinion towards genetic testing [39, 40], similar to the Dutch study [10], in our research the opposite results have been found. Although, it is worth highlighting that after the multivariate regression model was applied in the Dutch study the significance diminished, which goes in line with the very low beta values detected in our analysis.

The finding that those who had previous genetic test within the nuclear family (self, partner, children) have a more negative opinion on both the personal benefits and the usage of genetic testing could be explained with the hype around genetic testing [41]. After the Human Genome Project started there were high hopes that genomic technology will revolutionize healthcare as a whole. However, it is now clear that only in specific conditions does the genetic testing provide utility for the patients [2, 42]. Therefore, it is possible, that those who are familiar with genetic testing via personal experience realize the limitations of this approach, thus, have a more realistic opinion compared to the general population. Similarly, having a genetic test within the nuclear family (self, partner, children) could result in a better understanding how genetics impact the individual, which could lead to firmer belief in genetic determinism.

As expected from one of the Dutch studies [10], being religious resulted in a more negative opinion on both the personal benefits and the usage of genetic testing. This could be attributed to the notion that genetic testing raises moral and ethical questions which could be in conflict with some religious worldviews [24]. Finally, it is worth mentioning that we could not find any reasonable explanation in the literature why those being married believed more in genetic determinism. A possible answer could be that the level of education of the spouse might have influenced the attitude of the responder as well.

Previous surveys have already demonstrated that when comparing the citizens' attitude of various countries on the topic of genomic testing and genomic technology a noteworthy difference can be observed [12, 26]. However, in such cases it was unclear how the characteristics of the respondents might have influenced the results. By considering various confounding effects, it could be seen in our study that significant differences in opinions do exist between the citizens of the four countries. However, it is unclear what explanation could be given.

The study had many limitations worth highlighting. First, as indicated previously, due to the nature of ecological studies we could only identify and describe the differences, but not the reasons why do these differences exist. Second, citizens above the age of 65 were not involved in the analyses because the companies conducting the surveys did not include them in their database. Since higher age is associated with a more rejective attitude towards genetic testing [39, 40], the overall accepting opinion could be biased by this shortcoming. In the Dutch study which served as reference to the interpretation of our results in 2002 28%, while in 2010 36% was 65 years old and above. In addition, the level of education strongly increased in the Dutch population and reached a level which could only be observed in case of Hungarians among the populations of the V4 countries. However, this is most probably because the Hungarians with higher education were overrepresented in our study. Thus, it is also possible that the education level for the other three countries were also not representative.

Third, although the results of the confirmatory factor analysis regarding the four translations were close to the recommended thresholds, nearly none of these met the established criteria [35–37]. Because the original Dutch studies did not conduct such analysis [10, 25], it is unsure if this is a limitation of the instrument or if the four translations were inadequate. Fourth, the questionnaire used in our study does not differentiate the various types of genetic testing, nor takes the used circumstances into account. Thus, it is unclear what elements of genetic testing does the respondents agree or disagree with. Finally, the questions used in our analyses only focused on a small area that can be relevant on determining the citizens attitudes. Topics that are deemed by experts as important, such as the potential impact on discrimination and unequal access to such technology, were not addressed in this study [43, 44].

However, besides these limitations, there are several strengths that are also worth mentioning. First and foremost, the study had a large sample size from all four countries which was representative not only by age and sex but the number of residents living in the settlement and the regions were also considered. Thus, surveys in all the four countries could be deemed as comprehensive at a national level. Second, the questions adapted were proven to be reliable in the Dutch studies [6, 10, 25, 32], and with the strong internal consistency we can assume that this same reliability is present in our study as well. Finally, by including sex, age, education level, marital status, religiousness, and having a genetic test in the past as confounding factors, these as possible factors could be excluded from explaining the differences between the four countries.

## Conclusions

Overall, this study has demonstrated that noteworthy differences exist between the Visegrad countries regarding the citizens’ attitude towards genetic testing. As this research cannot explain the underlying reasons behind the differences, future studies should investigate this question more thoroughly. In order to gain a more precise understanding we recommend that these studies specify the type and purpose of genetic testing, the circumstances these tests are performed, and the possible underlying health conditions of the respondents. Furthermore, an in-depth comparison between Hungary and Slovakia would be highly recommended as the differences were most noteworthy between these two countries.

Although citizens become more open to genetic technologies as time passes [25], and despite the relatively accepting attitude identified in the Visegrad countries, policy makers should take further action to inform their citizens on the potential benefits of using genetic testing in medicine and public health and to address the concerns as well. This way it could be ensured that the citizens will perceive genetic testing as an integral part of health services, and thus facilitate a more personalized prevention and treatment as well. In addition, since genetic counsellors will be the healthcare professionals who will most likely communicate with the patient undergoing a genetic test [45], we recommend that these experts give more attention to patients who have higher education and are religious, as these groups are more sceptic on the personal benefits and the usage of genetic testing.

## Abbreviations

CFI: Comparative Fit Index
L.P.: Limited partnership
Ltd.: Limited company
RMSEA: Root Mean Square Error of Approximation
SD: Standard deviation
SRMR: Standardized Root Mean Square Residual
TLI: Tucker-Lewis Index
V4: Visegrad countries
